# Prospects in Innate Immune Responses as Potential Control Strategies against Non-Primate Lentiviruses

**DOI:** 10.3390/v10080435

**Published:** 2018-08-17

**Authors:** Lorena de Pablo-Maiso, Ana Doménech, Irache Echeverría, Carmen Gómez-Arrebola, Damián de Andrés, Sergio Rosati, Esperanza Gómez-Lucia, Ramsés Reina

**Affiliations:** 1 Instituto de Agrobiotecnología (IdAB), UPNA-CSIC-Gobierno de Navarra, Navarra 31192, Spain; lorena.depablo@unavarra.es (L.d.P.-M.); irache.echeverria@unavarra.es (I.E.); carmen.gomez@unavarra.es (C.G.-A.); damian.deandres@csic.es (D.d.A.); 2 Dpto. Sanidad Animal, Facultad de Veterinaria, Universidad Complutense de Madrid, Madrid 28040, Spain; domenech@ucm.es (A.D.); duato@ucm.es (E.G.-L.); 3 Malattie Infettive degli Animali Domestici, Dipartimento di Scienze Veterinarie, Università degli Studi di Torino, Torino 10095, Italy; sergio.rosati@unito.it

**Keywords:** non-primate lentivirus, control strategies, innate immunity, restriction factors

## Abstract

Lentiviruses are infectious agents of a number of animal species, including sheep, goats, horses, monkeys, cows, and cats, in addition to humans. As in the human case, the host immune response fails to control the establishment of chronic persistent infection that finally leads to a specific disease development. Despite intensive research on the development of lentivirus vaccines, it is still not clear which immune responses can protect against infection. Viral mutations resulting in escape from T-cell or antibody-mediated responses are the basis of the immune failure to control the infection. The innate immune response provides the first line of defense against viral infections in an antigen-independent manner. Antiviral innate responses are conducted by dendritic cells, macrophages, and natural killer cells, often targeted by lentiviruses, and intrinsic antiviral mechanisms exerted by all cells. Intrinsic responses depend on the recognition of the viral pathogen-associated molecular patterns (PAMPs) by pathogen recognition receptors (PRRs), and the signaling cascades leading to an antiviral state by inducing the expression of antiviral proteins, including restriction factors. This review describes the latest advances on innate immunity related to the infection by animal lentiviruses, centered on small ruminant lentiviruses (SRLV), equine infectious anemia virus (EIAV), and feline (FIV) and bovine immunodeficiency viruses (BIV), specifically focusing on the antiviral role of the major restriction factors described thus far.

## 1. Introduction

Lentiviruses are retroviruses that induce slow-developing diseases commonly unnoticed for several months or even years, until the final clinical stages that inexorably lead to the individual’s death. In spite of tremendous efforts aiming at controlling lentiviral infections in the last 40 years, a definitive strategy to limit the spread of the infection has not yet been developed [1]. Antiretroviral treatment is the only strategy able to maintain a healthy status of the infected individual, although economic issues restrict this strategy to humans. Promising vaccination strategies are being devised constantly. However, sterilizing immunity has not been reached, due to viral heterogeneity and escape mutants selection. A logical strategy has consisted in unveiling humoral and cellular adaptive responses as it correlates to protection after infection. Lately, the innate immune response on behalf of understanding basic processes capable of restricting lentiviral replication, has become a realistic alternative to control lentivirus spread [2].

**Figure 1:**
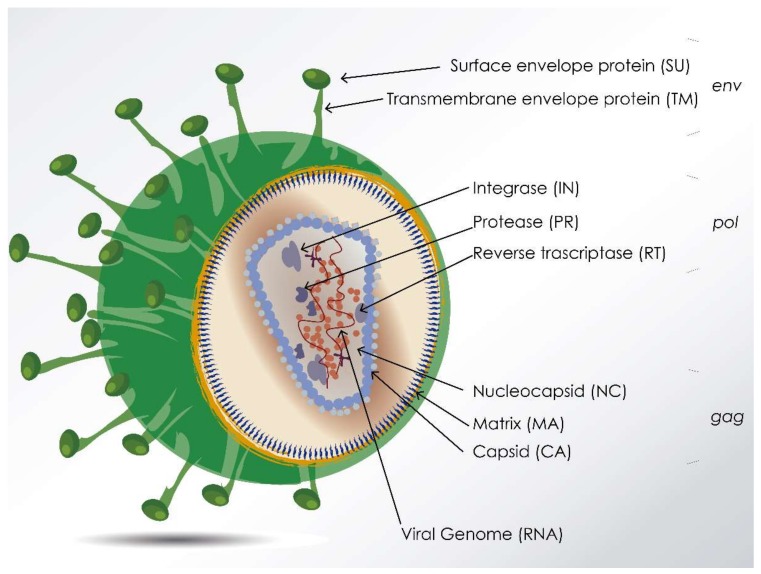
Virion particle schematic representation of animal lentiviruses. Figure depicts common structural genes and encoded proteins. Gene *gag* encodes the viral core proteins, including capsid (CA), matrix (MA), and nucleocapsid (NC) proteins. CA is relatively conserved, NC directly interacts with viral RNA. Gene *pol* encodes the replication-related enzymes, such as protease (PR), reverse transcriptase (RT) that catalyzes the viral DNA synthesis, and integrase (IN). Gene *env* encodes the envelope polyprotein that, after digestion by a cellular protease, result in transmembrane (TM) and surface (SU) subunits.

All lentiviruses generally share common features regarding virion particle structure (Figure 1), genetics, infection kinetics, tropism, immune response elicited, transmission, etc. In general, lentiviruses infect immune cells (mainly macrophages and T lymphocytes) and induce several types of immune dysfunctions (macrophage subset differentiation, T-cell anergy, etc.), inflammation, and organ/tissue impairments. 

Innate immune response is elicited early after infection onset, and contrarily to adaptive immune response, it does not require the specific recognition, processing, and presentation of the infective agent (antigen presentation) to trigger a response. The innate immune response is mediated by broad spectrum interactions between pathogen associated molecular patterns (PAMPs) and germline-encoded pathogen recognition receptors (PRRs) [3]. After engaging, normally on the surface of immune cells (mostly macrophages and dendritic cells), intracellular signaling induces a series of downstream events intended to face the infection. Among the multiple blocks supplied by the innate immune response, intrinsic factors, such as interferon-stimulated genes (ISGs), are gaining increasing interest in animal as well as primate lentivirus research, due to efficient inhibition of the viral replication cycle at multiple steps. Among ISGs, the focus has been put on a handful of molecules including TRIM5α, APOBEC3, and tetherin (BST2). TRIM5α is able to recognize viral incoming capsid in the pre-integration complex, and induces a premature decapsidation, impairing integration and significantly reducing proviral load and viral production, after auto-ubiquitination and autolysis in the proteasome. APOBEC3 is a cytidine-deaminase that causes uracil accumulation in negative-strand nascent DNA, leading to detrimental G to A mutation in the proviral DNA. Lastly, tetherin is able to trap virions inside the cells, avoiding virus budding, and therefore, virus transmission between cells. Lentiviruses are quite species-specific, and this innate immune response is responsible for preserving the species barrier. Thus, intrinsic restriction exerted by innate immunity would be able to block the infection by a lentivirus from other species [4].

On the other hand, evolution has shaped viral genetics in order to evade these restriction mechanisms. For example, in HIV infection, the viral protein Vif has the main function of avoiding APOBEC3 incorporation into viral particles. Vpu is particularly dedicated to dock tetherin and degrade it via proteasome, and the high genetic heterogeneity found within the capsid region is likely a consequence of long-lasting direct contact with TRIM5α proteins. This so-called “evolutionary arms race” has led to a strong purifying selection in somatic genes encoding virus-interacting proteins. So far, these innate mechanisms have been partially unveiled in the simian or human counterparts. However, recent research involving equine, ovine, bovine, and feline lentiviruses has been conducted.

Among lentiviruses affecting animal production, those infecting equines (EIAV) and small ruminants (SRLV) are the most important in terms of economic losses. *Feline immunodeficiency* virus (FIV) is a big concern for veterinarians who have to advise cat owners in the absence of treatments. On the other end of the spectrum, infection in cattle by bovine immunodeficiency virus (BIV) is usually a laboratory finding, as it is generally asymptomatic, although a related bovine lentivirus, *Jembrana disease* virus (JDV) causes severe and acute disease in Bali cattle. The comparison of the host responses against these viruses may shed some light on how to fight the most virulent ones.

In this review, recent advances in the control of non-primate lentiviruses based on early diagnosis, vaccination, and innate immunity are compared, with special attention to the intrinsic restriction factors, envisioning new strategies oriented to control lentivirus occurrence. 

## 2. Prevention and Control

Despite great efforts in controlling lentiviral infections through vaccination, mostly after the discovery of HIV, no satisfactory immunization strategy has been found. Lentivirus infection is difficult to control, due to the virus’ ability to integrate into the host’s genome, inducing persistent infections and eluding the immune response. However, animal lentiviruses can be controlled through different strategies, including early diagnosis and animal management or vaccination with attenuated viruses.

### 2.1. Small Ruminant Lentiviruses (SRLV)

**Figure 2:**
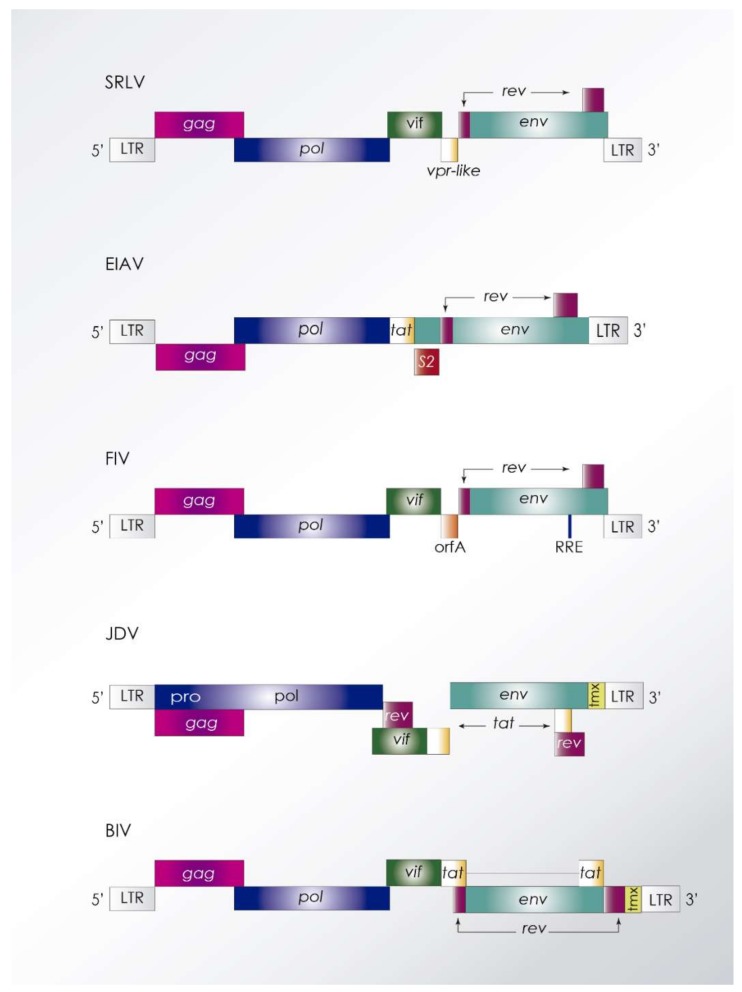
Proviral genomic structure of most common animal lentiviruses. Genes *gag*, *pol*, and *env*, as well as flanking long terminal repeats (LTRs), are common features among animal lentiviruses. Small ruminant lentiviruses (SRLV) encode three accessory genes *vif*, *vpr*-like, and *rev* without evidence of a transactivation activity [8]. *Equine infectious anemia* virus (EIAV) encodes an *S2* gene whose protein may accomplish a function similar to that exerted by HIV Nef [9]. *Feline immunodeficiency* virus (FIV) encodes *orfA* with functions similar to Vpr and Nef, since it produces G2 cell cycle arrest and downregulation of E2 ubiquitin conjugating enzymes [10], respectively. *Jembrana disease* virus (JDV) and *bovine immunodeficiency* virus (BIV) encode four accessory genes, *vif*, *tat*, *rev*, and *tmx* that may exert Nef-like properties. Animal lentiviruses also encode a dUTPase subunit involved in regulating cellular dNTP ratio. BIV encodes a dUTPase-related gene without enzymatic activity.

The *Visna maedi* virus (VMV) (the first lentivirus to be isolated) and the *Caprine arthritis encephalitis* virus (CAEV) belong to the small ruminant lentiviruses (SRLV) group [5] (Figure 2). Despite being initially considered as different entities infecting sheep and goats, respectively, molecular epidemiology studies have shown that the same variants are clearly present in sheep and goats in the field, thus, leading to natural cross-species infection. SRLV infection is widely distributed, causing slow multisystem and progressive inflammatory lesions in different organs, exerting meningoencephalitis, arthritis, pneumonitis, and/or mastitis [6]. Due to the slow and progressive course of the diseases produced by these viruses, listed by the OIE (World Organisation for Animal Health), the infection is frequently underestimated in a given population. To date, four genotypes have been described, with genotypes A and B hosting sequences from classical VMV and CAEV, respectively. Genotypes C and E have been described as geographically restricted to Norway and Italy, respectively. Molecular information on new subtypes is continuously updated with marked intensity in the last years showing a high genetic and antigenic heterogeneity.

The high genetic diversity of SRLV hinders the development of effective vaccines, and currently, there is no effective vaccine to prevent the infection by SRLV, despite intensive research on this topic [7]. Consequently, control strategies are exclusively based on diagnostic tools, relying on the identification of infected animals by early diagnosis, and the isolation of identified animals and their offspring. Diagnosis of SRLV infections cannot be made clinically, since only a small proportion of animals develop clinical signs, and in some cases, it can take months or years after infection for clinical signs to show up. Serological diagnosis is, to date, the most used method, but important drawbacks, mainly due to delayed seroconversions and the mismatching between circulating strains and diagnostic tests, have prompted the development of PCR methods.

Specific antibodies induced after natural and experimental infection have been detected by methods such as agar gel immunodiffusion (AGID) and enzyme-linked immunosorbent assay, among others. ELISA is now considered the most sensitive serological test. However, a ‘‘gold standard’’ method of diagnosis has not been established, and some studies have described the presence of viral sequences amplified by PCR from blood or tissues of seronegative animals. A combination of ELISA and PCR might afford optimal detection of SRLV infection [1,11].

Selection and breeding of resistant animals from the genetic point of view is of particular interest in viral diseases without treatments or effective vaccines. Ovine GWAS (genome-wide association studies) identified a transmembrane protein gene, *TMEM154*, as a candidate gene for SRLV resistance. Later, allelic differences in the *TMEM154* locus were associated with SRLV infection, and proposed as a locus for genetic marker-based selection [12]. Sheep with the ancestral *TMEM154* haplotype mutation were highly susceptible compared to sheep homozygous for missense mutation. A SNP linked to *TMEM154* indicated that the Dalsebred, Herdwick, and Rough Fells breeds from the United Kingdom have a low risk of SRLV infection [13]. Another transmembrane protein, TMEM38A, has been also identified as a gene associated with SRLV resistance, whereas the *DPPA2* gene was associated with susceptibility to SRLV infection [14]. However, TMEM154 function is unclear, and selecting towards an unknown character may represent more drawbacks than solutions. Virus adaptation may occur very rapidly if the interaction is not essential or if other host proteins may mimic TMEM154 function.

Deletions in chemokine (C–C motif) receptor 5 (*CCR5*) promoter have been linked with differential expression and reduced proviral levels of SRLV [15], and *CCR5* gene expression was upregulated in animals with lung lesions [16]. However, Alshanbari and colleagues [17] did not find a consistent association between SRLV proviral load and the *CCR5* promoter deletion.

Future discoveries and advances in understanding the biology of SRLV–host interactions are essential in identifying means to control viral transmission, the disease manifestation, or both.

### 2.2. Equine Infectious Anemia Virus (EIAV)

Equine infectious anemia is a chronic disease first described in France in 1843, and was shown to be caused by *Equine infectious anemia* virus (EIAV) in 1904 [18]. Worldwide distribution of this infection is challenging the horse industry, being one of the eleven notifiable equine diseases listed by the OIE.

EIAV is the lentivirus hosting the simplest genome including, along canonical structural genes, only three additional genes *tat*, *rev*, and *S2* (Figure 2). EIAV induces a disease with variable course in infected horses (acute, chronic, and unapparent) being viral and host factors involved in determining this clinical outcome. Viral factors essentially include antigenic variation to escape immune response and host factors, include age, immune competence, and MHC haplotype [19].

EIAV affects horses, mules, and donkeys with different virulence. While horses suffer chronic and acute disease, donkeys are asymptomatic, and present lower viral loads, suggesting the presence of natural restrictive mechanisms [20]. Main clinical manifestations consist of fever and thrombocytopenia [18]. One year post-infection infected animals typically progress to life-long unapparent carriers, but continue to face low levels of viral replication in monocyte-rich tissues [21]. Stress or immune suppression can induce peaks of viral replication, and potentially, a reactivation of the disease. Despite high virus replication and rapid antigenic variation, more than 90% of infected animals progress from chronic disease to an asymptomatic carrier stage, accomplished through tight immunologic control [22]. These animals are resistant to subsequent virus exposure, suggesting the development of proper protective responses and identifying critical immune correlates of protection.

The biology of EIAV is perhaps the most unique among lentiviruses from various points of view. Unlike most other lentiviruses, EIAV is transmitted through a winged insect (horseflies and deerflies), and the animal is able to control the infection by producing neutralizing antibodies. However, sterilizing immunity is not reached, and the infection persists lifelong with acute and chronic periods, related to the re-emergence of neutralization escape mutants, mainly in the Env region [21].

The control of EIAV infection relies on early diagnosis and vaccination depending on the geographical region considered. In Europe, the infection is diagnosed through agar gel immunodiffusion (AGID) test (Coggin’s test). As for other lentiviral infections, early detection is hampered by the presence of a low viral load that elicits low antibody titers, integration into host’s genome jeopardizing antigenic exposure, and divergence between tests and field strains. Despite being generally limited to research studies, molecular detection methods have also been applied through PCR, measurement of RT activity, or ELISA using monoclonal antibodies [23]. In this regard, a new phylogenetic spectrum of strains has been uncovered recently in Europe [24], challenging the development of new, highly sensitive serological tools. Up to 17% of the infected horses are misdiagnosed using AGID [25,26,27]. PCR-based procedures have provided a higher diagnostic power. However, until recently, only a few EIAV isolates had been completely sequenced: North American Wyoming, Chinese Lianing, Japanese Miyazaki, and the European strain identified in Ireland, IRE. The subsequent phylogenetic analysis showed that the genetic space occupied by EIAV strains is much wider than previously thought, significantly increasing the knowledge of molecular epidemiology that turns out to be essential in designing molecular diagnostic tools [28]. Recently, next generation sequencing (NGS) has drastically increased the available information on genetic variation within EIAV phylogenetic space [24]. 

From the early 1970s, an EIAV vaccine had been developed based on attenuated viruses via *in vitro* passages, obtaining a vaccine strain EIAV DLV120. This vaccine was extensively used in China, controlling disease occurrence in more than 60 million horses and suggesting that attenuated viruses can be safely used in the control of animal lentiviral infections [29]. Viruses harboring artificial mutations in the *S2* accessory gene have also resulted in effective attenuation and protection against homologous challenge (EIAVd9). However, protection was inversely correlated with antigenic heterogeneity in the envelope proteins, suggesting potential selection of escape mutants under immune pressure [30].

### 2.3. Feline Immunodeficiency Virus (FIV)

*Feline immunodeficiency* virus (FIV) was first isolated in California in 1986 from a colony of domestic cats seronegative for the *Feline leukemia* virus (FeLV) that exhibited immunodeficiency [31]. FIV infection is present worldwide with an estimated seroprevalence of 11.04%, though it may vary greatly between countries [32]. The infection is transmitted mostly by the direct inoculation of viruses in the saliva through bite wounds during fights or mating courtship. Therefore, prevalence is higher in adult males than in females, and in cats who have been allowed to roam freely, while transmission between cats in stable households is much less common. FIV can also be transmitted vertically through milk and colostrum [32].

FIV has been diagnosed in at least 11 felid species, representing the most well-defined lentiviral group outside the *Simian immunodeficiency* viruses (SIVs) with interspecies infection ability. Though lentiviruses exhibit a high degree of species specificity, FIV cross-species transmission has been widely described also in Felidae, mostly as a singular event, with the exception of repeated transmissions of FIVs from bobcats to pumas [33].

Based on nucleotide sequence diversity of the envelope gene, FIV can be classified into five subtypes [34]. Eighty percent of FIV viral isolates worldwide belong to the A or B subtypes, and evidence suggests that subtype B is the oldest [35]. FIV shares many characteristics with HIV, including infecting and replicating primarily in CD4+ T-cells following a progressive depletion of these cells, leading to immunosuppression that allows opportunistic pathogens to infect and contribute to an AIDS-like syndrome. Other target cells are B-cells, macrophages, and dendritic cells [36]. After an initial set of non-specific clinical signs, coinciding with the period of initial viremia, which reaches its maximum 8–12 weeks post-infection, infected cats may be asymptomatic for many years. Non-specific clinical signs—such as cachexia, gingivitis-stomatitis, wasting, and neuropathological disorders—develop, together with an increased incidence of malignancy, which is specifically referred to as the feline acquired immunodeficiency syndrome (FAIDS).

Viral replication rate and the clinical signs seem to vary between different strains [37]. With proper care, FIV-infected cats can live many years and, in fact, can die at older ages from causes unrelated to their FIV infection.

Classical virus detection by blood cell culture and virus isolation from plasma or peripheral blood lymphocytes is possible over the whole infection period, but not practical from a routine diagnostic point of view [38]. FIV infection is currently diagnosed by antibody detection in the blood. Antibodies usually appear within 2–4 weeks after experimental infection, and eventually are detectable for the rest of the life [39]. However, a small portion of experimentally infected cats may not present antibodies for up to 1 year following infection. Furthermore, in the final stage, antibody levels can fall below the detection level [38]. In practice, rapid tests have been developed for the diagnosis of FIV infection, generally combined with the diagnosis of FeLV infection. Most of these tests use an immunochromatography-based assay using whole blood, serum, plasma, or even saliva and labeled anti-cat IgG and FeLV-p27 antibodies in a small device (dipstick).

A vaccine was licensed in the United States in 2002, including the inactivated subtype A (Petaluma strain) and subtype D (Shizuoka strain) [40], but not a representative strain of clade B, prevalent in many parts of the United States. The initial dose is recommended to be administered to kittens as early as 8 weeks of age, with two subsequent booster doses at an interval of 2–3 weeks [41]. No FIV vaccine has been licensed in Europe, since the American vaccine does not allow the differentiation of vaccine derived from infection-derived antibodies.

### 2.4. Bovine Lentiviruses

*Bovine immunodeficiency* virus (BIV) and *Jembrana disease* virus (JDV) are two closely related lentiviruses that infect bovids. BIV was first identified in the 1960s from a dairy cow (named R-29) (*Bos taurus*) in the United States, with persistent lymphocytosis, neurological disorders, and a progressive wasting syndrome [42]. Initially, the virus was named “*bovine visna-like* virus”, but due to the high similarity to HIV, it was finally designed as “*bovine immunodeficiency* virus”. JDV was described in the 1990s from an acute disease in Bali cattle (*Bos javanicus*) [43].

BIV is distributed worldwide (serological and molecular lines of evidence have been reported in many countries) [44], whereas JDV is endemic to parts of Indonesia [45].

It has been proposed that BIV is a lentivirus that has co-evolved with its host to the point that usually causes asymptomatic infection. On the contrary, JDV has probably arisen from BIV via an unknown transmission event, and causes an acute, and sometimes lethal, infection in a specific bovine species (Bali cattle) [45]. This increased pathogenicity could be related to the species barrier transgression, as it has been described previously with other lentiviruses. Highly likely, cellular restriction factors are able to block retroviral replication in the host (*Bos taurus*), as will be seen later in this review. In spite of these different clinical outcomes, BIV and JDV are molecularly and antigenically related, and cannot be differentiated serologically with immunodiagnostic methods [43].

Despite causing mild disease, several clinical signs have been described in BIV-infected cattle, such as lymphocytosis, lymphadenopathy, neurological disorders, progressive weight loss, diminished milk production, and secondary infections (probably due to immunosuppression) [46,47]. Although clinical disease has not been fully reproduced in cattle, there is evidence suggesting that BIV can cause immune disturbance [48]. Other studies have demonstrated a fatal immune dysfunction in rabbits experimentally infected with BIV, similar to that developed in cats inoculated with FIV [49].

On the contrary, JDV causes an acute and severe disease with high levels of viremia. Clinical signs include fever, lethargy, anorexia, lymphadenopathy, and lymphopenia; the mortality is approximately 15–20%. There is no recurrence of disease in recovered animals. JDV is often described as an atypical lentivirus, because of the low level of viral variation *in vivo*, the delayed antibody response, and the ensuing immunological control which prevents subsequent heterologous infection [45].

BIV is pathologically more related to lentiviruses associated with chronic inflammatory diseases (SRLV and EIAV) than those associated with severe immunodeficiency (HIV, FIV, and SIV) [50]. Like EIAV, transmission is probably via hematophagous arthropods, such as tabanid flies, during the acute phase of disease, with high levels of viremia, although other routes of transmission are also possible [45].

The non-pathogenic nature of BIV, despite its close genetic and antigenic similarity with pathogenic lentiviruses like JDV and HIV, is an interesting feature that makes this virus a good model for lentiviral research [46].

An inactivated tissue-derived vaccine against JDV is used to control the disease in Indonesia. This vaccine reduced the duration and severity of the disease, and the level of viremia during the early febrile and recovery periods, decreasing the risk of virus transmission to other cattle [51]. 

## 3. Innate Immunity

The innate immune system provides a first line of defense against viruses. Dendritic cells and macrophages play key roles in initiating and coordinating this first-line immunity with the adaptive immune response. However, in several animal lentivirus infections, macrophages also represent the main target cell, which probably causes immune dysfunction. Soon after viral infection, cells of the innate immune system use pattern-recognition receptors (PRRs) to sense pathogen invasion by engaging pathogen-associated molecular patterns (PAMPs). It has been proposed that a “trinity” of pathogen sensors operate the innate immune response to pathogen infection: the nucleotide binding oligomerization domain-like receptor (NLRs), the retinoic acid-inducible gene (RIG)-like receptor (RLRs), and the Toll-like receptors (TLRs) [52]. NLRs and RLRs have not being studied, and little information on the possible role of TLRs concerning animal lentiviral replication and pathogenesis is available.

**Figure 3:**
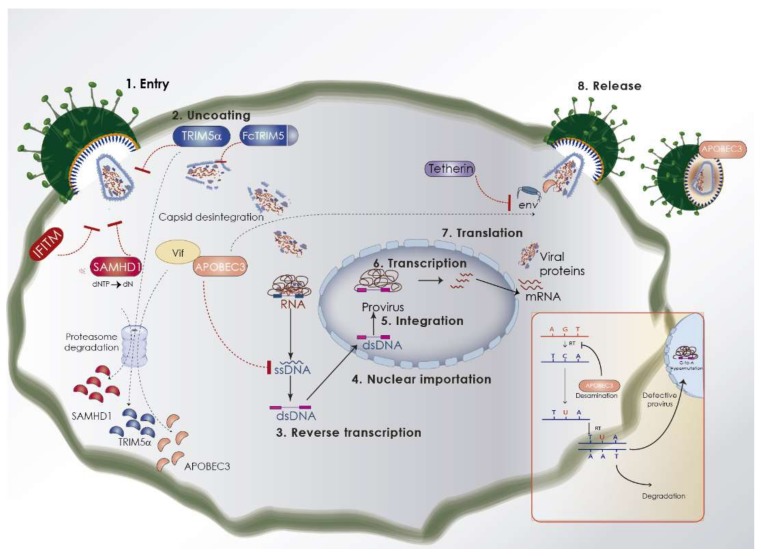
Overview of the lentivirus replication cycle and cellular restriction factors that target lentiviruses and their viral antagonists. The key mechanisms by which restriction factors directly act upon the retroviral replication cycle, and their counteraction by viral accessory proteins, are depicted. Virus replication steps are shown with numbers. After entry, TRIM5α recognizes viral capsid and induces premature decapsidation and autoubiquitination, except in the case of *Felis catus*. Other measures that may inhibit the infection cycle at this stage are SAMHD1 and IFITM (interferon-induced transmembrane protein 3). Viral Vif can counteract some of these mechanisms by inducing proteasome-associated degradation. APOBEC3, which is harbored in viral particles, mediates hypermutation of the viral genome, as indicated in the inset panel. When the lentiviral genome and proteins are assembling, tetherin can trap new virions and reduce infectivity.

Engaging of PAMPs rapidly induces type I interferon (IFN-I) and pro-inflammatory cytokine synthesis. Induction of IFN-I regulates the expression of several “interferon stimulated genes” or ISGs whose protein products have direct antiviral properties. This group of proteins are also known as “restriction factors” and inhibit virus replication by targeting specific steps in the viral cycle. High genetic variability in the restriction factors might also limit the spread and extent of infections and disease within the natural host population (intra-species transfer) (Figure 3).

Activation of macrophages with phorbol esters or certain cytokines stimulates gene expression of EIAV [53], SRLV [54], or FIV [55] initially related to the activation of cellular transcription factors binding the viral LTR regulatory region.

The interaction of innate immunity and non-primate lentiviruses is not well understood yet. Here, we summarize some important aspects of this immunity in each of the viruses included in this review (SRLV, EIAV, FIV, and BIV/JDV).

### 3.1. SRLV

The context in which the innate immune system first encounters a pathogen is perhaps, the most important step in determining the induction of a protective adaptive immune response. This also stands for SRLV infection and susceptible macrophages, which can adapt their transcription profile under the proper stimuli, to differentiate into resistant cells [56].

Mannosylated residues in the Env protein of SRLV are likely sensed by the mannose receptor (MR; CD206), a C-type lectin endocytic receptor expressed on macrophages and dendritic cells. MR exogenous expression renders non-permissive CHO cells, permissive to SRLV infection, and MR expression has been associated with lesion development *in vivo* [57,58]. However, the MR role as a receptor that enables virus infection, or rather, leads to a restrictive process, is unknown.

Recently, genome-wide association studies (GWAS) determined an antiviral role of the host cellular restriction factor known as interferon-induced transmembrane protein 3 (IFITM3), highlighting the importance of the innate response in the fight against SRLV [59].

In addition to soluble factors (cytokines and interferons), innate immune response is also dependent on different cell types. Infection of dendritic cells (DC) by HIV-1 is important in the development of protective immune responses, in the spread and persistence of virus, and in the immune dysfunction that characterizes AIDS [60]. DCs are infected by SRLV *in vivo* and *in vitro*, and are important for the transfer of MVV from the site of infection to lymphoid tissue not only carrying virus, but infected with replicating virus [61]. γδT cells are a unique set of unconventional T-cells that are present in a high number in sheep, goats, and other ruminants, involving approximately 70% of all lymphocytes in young animals [62]. These cells localize to mucosal surfaces where they function as primary responders by recognizing intact structures in an MHC-independent manner [63], and exerting lytic activity and secretion of proinflammatory cytokines. SRLV-infected animals showed an increased proportion of γδ lymphocytes compared to healthy goats, suggesting an important function in controlling SRLV infection [62,64]. Natural killer (NK) cells may recognize infected cells and virion particles through a number of mechanisms, including killer immunoglobulin-like receptor (KIR)-mediated recognition, degranulation, complement activation, antibody-dependent cell-mediated cytotoxicity (ADCC), and the production of IFN-γ, which serves to either kill infected cells, or modulate virus-specific immune responses [65]. The role of NK cells in SRLV infection has not been deeply investigated. However, given the importance of NK cells in HIV-1 infection, it is likely that they play an important role in the control of SRLV. A single description reports a depressed NK activity in infected goats that may contribute to the establishment of a persistent infection [66].

### 3.2. EIAV

EIAV infection is considered as a model for natural immunological resistance against lentivirus replication and persistence [67].

The oxidant/antioxidant equilibrium is affected by EIAV modifying glutathione peroxidase (GPx) and uric acid levels, both danger signals of the innate immunity [68].

After infection, equine macrophages produce IFN-I and the subsequent induction of hundreds of ISGs with antiviral activity. Viperin (virus inhibitory protein endoplasmic reticulum-associated interferon-inducible) is one of these ISGs that regulates virus replication by broad antiviral activity against influenza A, HIV-1, hepatitis C, West Nile virus, Dengue, and EIAV at multiple steps of viral replication [69].

### 3.3. FIV

FIV infects T-cells (CD4+ and CD8+), cells of the monocyte/macrophage lineage, and cells of the central nervous system, through an initial interaction of FIV Env with the receptor CD134 and subsequent binding to the CXCR4 chemokine receptor [70]. CD134, a member of the tumor necrosis factor and nerve growth factor receptor superfamily, is a costimulatory molecule abundantly expressed on activated lymphocytes, monocytes, macrophages, and dendritic cells.

FIV-infected cats have an innate immune defect that is reflected in impaired clearance of the infecting pathogen inoculated subcutaneously, suggesting DC dysfunction related to antigen presentation and migratory capacities, rather than to Treg induction as it occurs in HIV-1 infection [71].

### 3.4. BIV and JDV

BIV infects, *in vivo*, mainly monocytes, macrophages, and lymphocytes [72]. Neuropathic BIV strains probably infect macrophages and microglia in the central nervous system (CNS), as other lentiviruses [73]. *In vitro*, BIV is able to replicate in several types of cells, as fibroblast-like cells, establishing persistent infections in different primary cultures of bovine cells, from lung, brain, or spleen. BIV can infect and transcribe its genome in different subsets of PBMC (CD3+, CD4+, CD8+, γδT-cells, B-cells, and monocytes) [74].

The receptors for BIV or JDV have not been identified yet. There is evidence that BIV may utilize C–C chemokine receptor 5 (CCR5), the main coreceptor used by macrophage-tropic strains of HIV-1, for infection of cells *in vitro*. Wright et al. [75] described the blockade of CCR5 in fetal bovine lung cells, which caused a decreased virus expression. A similar effect was also shown in HIV-infected cells *in vitro*, which provided the initial evidence that HIV utilizes chemokine receptors as co-receptors for infection of cells.

The replication of BIV in monocytes/macrophages probably causes a dysfunction of the immune system. Thus, various important monocyte functions, such as superoxide anion release, phagocytic activity, and chemotactic responsiveness, are depressed in BIV-infected calves compared with non-infected control calves [49].

JDV cell tropism is not fully elucidated. Desport et al. [76] reported an apparent tropism for IgG-containing cells, with no evidence of infection of CD3+ T-cells or monocytes in tissues. However, large vacuolated cells, with a macrophage-like morphology containing viral antigen, were seen in the lung, although they could not be conclusively shown to be infected. Thus, the role of macrophages in JDV infection of Bali cattle remains unclear. This difference could also partially explain the fact that JDV has not been associated with neurological lesions, while the lentiviruses that infect macrophages are able to cause neurological disease.

## 4. TLR Signaling

**Figure 4:**
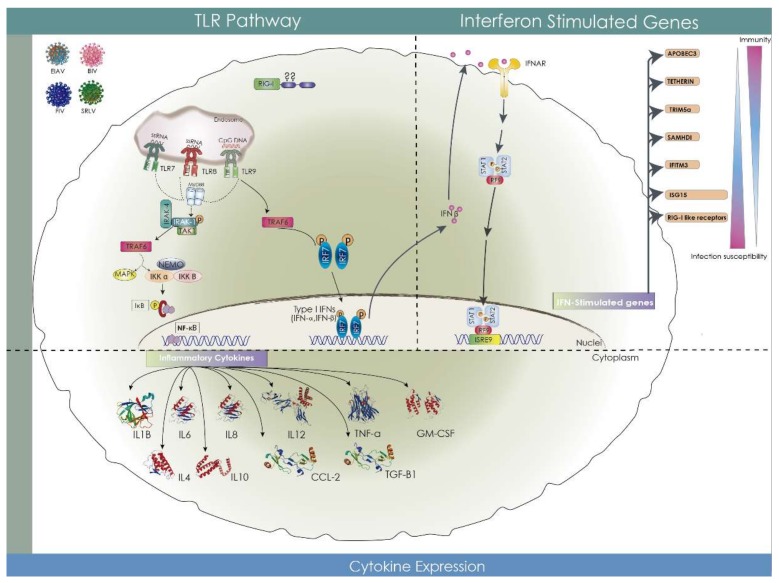
Animal lentiviruses innate immune sensing. Toll-like receptors (TLRs) 7, 8, and 9 can sense virus-derived nucleic acids and induce a signaling cascade involving IRAK-1 that results in the expression of inflammatory cytokines and type I-IFN, such as interferon alpha (IFN- α) and beta (IFN-β). Receptor for IFN (IFNAR) engages type I IFN molecules, inducing the expression of many antiviral proteins known as IFN-stimulated genes, among which, restriction factors are present.

Toll-like receptors are germline-encoded pattern recognition receptors (PRRs) that activate the innate immune system upon recognition of pathogens. They recognize structurally conserved molecules derived from invading pathogens (PAMPs) or endogenous damage signals (DAMPs). Various combinations of TLRs are expressed by different subsets of immune and non-immune cell types, such as monocytes, macrophages, dendritic cells, neutrophils, B-cells, T-cells, fibroblasts, endothelial cells, and epithelial cells. Some TLRs are expressed on the cell surface (and primarily recognize microbial membrane and/or cell wall components), while others are expressed in the membranes of endolysosomal compartments, and recognize pathogen-derived nucleic acids [52] (Figure 4). Recognition by TLR marks the key molecular events that ultimately lead to innate immune responses and the development of antigen-specific acquired immunity.

TLRs contain three domains, an N-terminal leucine-rich repeat (LRR) domain, a single transmembrane (TM) domain, and a C-terminal Toll/interleukin-1 receptor (TIR) homology domain [77]. The TIR domain mediates interactions between TLRs and adaptor proteins (proteins that mediate other protein–protein interactions) involved in regulating TLR signaling. These include myeloid differentiation primary-response protein 88 (MyD88), a TIR domain containing adaptor protein inducing IFN-β, TRAM, and TIRAP/MAL. The phosphorylation following activation allows NF-κB to translocate to the nucleus, and induces the expression of target genes such as pro-inflammatory cytokines, chemokines, and type I and type III interferons [78,79].

Although TLRs provide protection against a wide variety of pathogens, inappropriate or unregulated activation of TLR signaling can lead to chronic inflammatory and autoimmune disorders.

TLRs 3, 7, 8, and 9 play a crucial role in the recognition of viruses by targeting distinct types of virally-derived nucleic acids, and activate signaling cascades that result in the induction of type I IFNs. Viruses typically lack the conserved features common to other pathogens, so the innate immune system has evolved to recognize viral nucleic acids as a hallmark of viral infection (Figure 4). Sensing of viral nucleic acids by TLRs usually triggers the production of type I IFN [52], though activation may be dependent on the IRF-7 pathway [80]. This is the case of TLR9 signaling, which has become the focus of much attention in the regulation of adaptive immune responses [81].

Agonistic ligands of nucleic acids (NA)-sensing TLRs play an emerging role in the treatment of viral diseases, demonstrating a crucial role of these receptors. Recently, crystal structures have afforded new insights into TLR recognition of nucleic acids. As for other TLRs, an aberrant activation by self-genetic material leads to inflammation and autoimmunity that is generally avoided by strict regulation of NA–TLR interaction at multiple checkpoints [77].

The role of TLRs in the host defense against viruses makes them appropriate markers for the study of TLR-associated polymorphisms and susceptibility to viral infections [82].

### 4.1. SRLV

Activation of TLRs can have significant effects on adaptive immune responses. The mechanisms for innate immune recognition of pathogens and signaling have received special attention. However, the role of virus-induced TLR signaling has not been widely studied in sheep and goats. SRLV infection influences the expression of different cytokines in infected cells, and modulates the cytokine response after PRR stimulation. During SRLV infection, TLR7 and 8 become activated, inducing cytokines and expression of antiviral proteins [7], and are maintained significantly upregulated in animals with SRLV-induced lung lesions [16]. Activation of TLR7/8 would predominantly induce Th1 cytokines and chemokines in addition to increased expression of co-stimulatory molecules [83]. Mikula et al. [84] reported association between SRLV infection and mutation frequencies in the *TLR7* and *TLR8* genes.

TLR9 is highly polymorphic, but no implication in SRLV infection has been observed [16,85,86]. In spite of this, G520R mutation in TLR9 is associated with SRLV seropositivity, and might be used in studies investigating SRLV susceptibility or resistance in sheep [87]. 

### 4.2. EIAV

Equine TLR2, 3, 4, 5, 7, and 8 have been fully sequenced. The equine TLRs have a nucleotide identity of 65–77% with the human counterparts [79].

Equine monocyte-derived macrophages (eMDM) infected with EIAVFDDV13, a Chinese attenuated EIAV strain, are resistant to subsequent infection by a pathogenic strain, EIAVUK3. The expression of the soluble EIAV receptor sELR1, TLR3, and IFN-β was upregulated in eMDM infected with EIAVFDDV13 compared with eMDM infected with EIAVUK3. Stimulating eMDM with poly I:C resulted in similar resistance to EIAV infection as induced by EIAVFDDV13, and was correlated with enhanced TLR3, sELR1, and IFN-β expression. The knockdown of *TLR3* mRNA significantly impaired poly I:C-stimulated resistance to EIAV, greatly reducing the expression of sELR1 and IFN-β, and the level of resistance induced by EIAVFDDV13. These results indicate that resistance is at least partially mediated by the activation of TLR3 pathways [88].

### 4.3. FIV

Feline TLR1, 2, 3, 4, 5, 7, and 8 have been detected in different feline tissues, mostly in the mesenteric lymph nodes (TLR2, 3, 5, 7, and 8) and in the spleen (TLR1, 4). TLR7 and 9 were those most expressed TLRs in cell lines infected by FIV, which confirms that FIV infection can alter TLR expression [89]. Similar results were found after FIV challenge, reporting a significantly increased TLR7 expression, decreased TLR2 expression, and with variable effects on TLR6, 8, and 9 in mosttissues [90]. Activation of TLR4 by FIV triggers CD4+CD25+ T regulatory cells [91], which could further enhance FIV virulence, due to immune downregulation. These data indicate a role for TLRs in host response to FIV.

### 4.4. BIV

The complete coding sequence of TLR1-10 is already known, with a nucleotide sequence identity to human TLR genes of 67–77%, and 98% to the closely related nilgai (*Boselaphus tragocamelus*) or water buffalo (*Bubalus bubalis*) genes, respectively [79].

The variable expression of TLRs during BIV/JDV infection and the possible role in the evolution of the disease is still unknown.

## 5. Interferon

Interferons are a family of cytokines classified into three types. Type I interferons (IFN-I) include IFN-α, IFN-β, and several others. Ruminants express IFN-τ, an IFN-I produced by the ruminant trophoblast with an important role in gestation [92]. Type I interferons are induced early after virus infection, and mediate innate immune response. Many cells may secrete IFN-α/β, but dendritic cells are the main producers of IFN-α.

Type II interferon (IFN-II), also called the immune interferon, only includes IFN-γ that is mainly synthesized by T-cells and NK cells. IFN-γ plays an important role in innate and adaptive immune responses, but this review will focus on IFN-I that mainly acts at early stages after infection.

Viral dsRNA activates the pathways of NF-κB and IRF-3, the main factors that regulate the transcription of IFNs. These factors cross the nuclear membrane and bind the *IFN-β* gene promoter, forming an enhanceosome complex. Other co-activators and RNA polymerase aggregate with the enhanceosome, allowing the transcription of IFN-β that is secreted.

In the next amplification step, IFN-β binds the IFN-α/β heterodimeric receptor (IFNAR) in an autocrine and paracrine manner, and through dimerization, activates JAK proteins (Janus tyrosine kinases) and initiates a positive feedback loop that triggers the activation of ISGF3 (heterotrimer of STAT1, STAT2, and IRF9) and the expression of IRF-7. IRF-7 has been identified as the main regulator of IFN-I expression [93] in collaboration with IRF-3 [94] (Figure 4).

TLR signaling activates IRF-7 directly, triggering the secretion of high levels of IFN-α [95,96,97]. Phosphorylated IRF-7 and ISGF-3 cross the nuclear membrane and bind DNA regulatory cis-acting sequences, called interferon-stimulated response elements (ISRE), which have a consensus sequence AGTTTCNNTTTCNC/T [98]. This sequence is present in several interferon-stimulated genes (ISGs) that encode proteins with antiviral activity, such as restriction factors. Among ISGs, APOBEC3, TRIM5α, and tetherin are the most studied proteins induced by IFN, with the ability to block lentivirus infection. These antiviral proteins interrupt different points in the viral replication, depending on the type of infected cell and the virus [94]. For these reasons, IFN-I has been used with a variable degree of success in treating viral infections, including retroviral infections.

However, retroviruses may contain ISRE in their LTR [99], by which viral genome transcription is enhanced when an IFN-induction pathway is triggered.

### 5.1. SRLV

SRLVs weakly induce Type-I IFN [100], but the lentivirus-induced interferon (LV-IFN), a mixture of IFN-I and II, is produced in cell cultures infected *in vitro* with SRLV. LV-IFN inhibits the maturation of monocytes to macrophages, and thus, inhibits virus replication. LV-IFN also has a direct influence inhibiting viral replication in mature macrophages [101]. The analysis of the SRLV genome has identified a sequence compatible with the consensus sequence for ISREs, but located outside the LTR, so it cannot function as an ISRE (Gomez-Lucia and Domenech, unpublished observations). Macrophages infected with SRLV *in vivo* and *in vitro*, exhibit an anti-inflammatory M2 profile, suggesting viral adaptation to this particular cell subset [56].

### 5.2. EIAV

In horses, seven different classes in the equine IFN family, including eight genes of IFN-ω, six IFN-α, and four IFN-β, have been described [102] with IFN-α being broadly antiviral [103]. Equine IFN-α showed potent antiviral activity against *Vesicular stomatitis* virus (VSV) and EIAV in early and late stages of infection, respectively. Human tetherin was dramatically increased in IFN-α treated cells, suggesting a central role for tetherin in EIAV natural restriction [104].

### 5.3. FIV

FIV infection stimulates an IFN-I response since expression levels of IFN-α, -β, and -ω, as well as IL-15 and TLR3, 7, and 8 are induced in feline PBMCs challenged *in vitro* with FIV [105].

As for other viral diseases, IFN-I has been used for treating FIV infections. Most reports have been focused on IFN-ω, of which a feline recombinant molecule is commercially available (rFeIFN-ω; Virbagen Omega, Virbac). This molecule has been found to act as an immunomodulatory drug, stimulating the innate immunity, decreasing clinical signs and co-infections in naturally FIV-infected cats [106,107], but not viremia or cytokines [108]. This suggests that rFeIFN-ω acts *in vivo* mostly by potentiating the innate response, specifically reducing the pro-inflammatory stimulus. In another study, treatment with rHuIFN-α was found to greatly improve the general clinical status of naturally FIV-infected cats, as well as clinical parameters, such as hematocrit, red blood cell counts, and white cell counts. The CD4:CD8 ratio and proviral load in circulating PBMCs were also improved. However, most of the parameters reverted to the original values when treatment with IFN-I was suspended [109].

In order to determine whether the positive effects seen in IFN-I treated cats were due to the enhancement of the innate immune response or to the virus itself, studies were undertaken, *in vitro*, on FIV-infected cells exposed to IFN-I (recombinant human IFN-α or recombinant feline IFN-ω). IFN-I treatment did not affect virus protein synthesis, which suggests that there are no functional ISRE in FIV. Contrariwise, treatment with IFN-I decreased the expression of FIV RT in a dose-dependent manner [109], which results in a lower amount of circulating infective particles. Thus, IFN-I helps to control FIV infection, not only by providing an antiviral state of the cells, but also by reducing the amount of infective viral particles [106].

### 5.4. BIV

As mentioned previously, binding of IFN-I to the cell receptors can induce the expression of several ISGs, many of which play a role in cellular innate immunity against viral infections. ISG15, one of the most strongly induced proteins by IFN, is a 15-kDa ubiquitin-like protein that can be covalently conjugated to target proteins for post-translational modification [50]. ISG15 has been reported to inhibit HIV-1 release by disrupting the interaction of the Gag L domain with tumor susceptibility gene 101 (Tsg101). Tsg101 is one of the cellular proteins involved in the budding process of HIV-1, with an important role in the cellular vacuolar protein sorting (Vps) pathway [50,110], and may play a role in the response against non-primate lentiviruses.

A similar bovine ISG15 (bISG15) has also been reported to inhibit BIV replication in fetal bovine lung (FBL) cells. Liu et al. [50] proposed that infection may activate interferon or NF-κB signal pathways, inducing bISG15 expression directly or through IFN-I.

## 6. Host Restriction Factors

Among the ISGs that can directly interfere with different stages of the virus replication cycle, Tripartite motif-containing protein 5 alpha (TRIM5α), apolipoprotein B mRNA-editing enzyme, catalytic polypeptide-like 3 (APOBEC3) and tetherin (also known as bone marrow stromal antigen 2, BST2) are upregulated by IFN-I, and have received much attention in the last few years.

The connection of intrinsic resistance mechanisms in controlling host restriction has been clearly shown for the primate lentiviruses, but it is not completely understood in non-primate lentiviruses [111]. However, the plasticity of lentiviruses allows them to counteract these restriction factors through their accessory genes.

### 6.1. TRIM5α

Tripartite motif-containing protein 5 alpha is a member of the tripartite motif protein family that binds viral capsid lattices, inducing proteasome dependent degradation, thereby inhibiting integration and post-integration steps of the virus replication cycle. HIV replication in simian cells is blocked during uncoating at early post-entry stages, a finding that was used to identify TRIM5α as a restriction factor, which is constitutively expressed, but interferon treatment can further increase expression levels [112].

**Figure 5:**
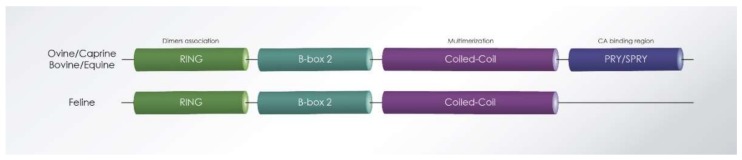
Schematic representation of ovine, caprine, bovine, equine, and feline Tripartite motif-containing protein 5 alpha (TRIM5α). RING, B-box-2, coiled-coil, and PRY/SPRY domains are represented. Feline TRIM5α lacks the PRY/SPRY domain because of a premature stop codon in the mRNA transcript. Predicted structure of GenBank accession numbers: JN835300-JN835311 (ovine) and JQ582845-JQ582849 (caprine), DQ380509 (bovine), XM_014741762.2 (equine), NM_001163659 (feline).

All TRIM proteins contain an N-terminal RING domain with E3 ubiquitin ligase activity that also assists the higher-order association of TRIM5α dimers, which promotes capsid binding [113]; one or two B-box domains, and a coiled-coil domain (RBCC) [114] (Figure 5). The TRIM5α isoform, which is active against retroviruses, also contains a C-terminal PRYSPRY domain. The PRYSPRY domain binds retroviral capsid (CA) of incoming viral particles [115], while the RBCC mediates the localization to cytoplasmic bodies and is important for TRIM5α self-association [112]. The PRYSPRY domain determines viral specificity and restriction potency, and sequence variations in this domain correlate with the ability of TRIM5α to recognize different viral capsids [116]. Multimerization through the coiled-coil domain increases TRIM5α avidity for viral capsids, which further potentiates antiviral restriction [117].

Upon recognition of viral incoming capsid present in the pre-integration complex (prior to integration), TRIM5α suffers auto-ubiquitination and autolysis, inducing a premature decapsidation. Consequently, integration and proviral load are reduced. Recent electron microscopic studies [118] have demonstrated that purified TRIM5α proteins spontaneously form a large hexagonal lattice structure on the HIV-1 capsid, which is composed of smaller CA hexameric units. Interactions between TRIM5 proteins and viral capsids promote the uncoating of sensitive viruses [113]. Species-adapted retroviruses evolved viral capsids that elude TRIM5α proteins expressed in their host species [119], giving rise to the concept of heterologous restriction, by which restriction factors encoded by a given species would better inhibit replication of lentiviruses affecting other species [120].

Recognition of the retroviral capsid by TRIM5α has been shown to promote innate immune signaling by catalyzing the synthesis of unattached K63 ubiquitin chains that activate TAK1 kinase. TRIM5α has a specific effect on the expression of NF-κB- and AP-1-responsive inflammatory chemokines and cytokines, and plays a role in LPS-triggered immune activation through the TLR4 pathway [121].

The first non-primate TRIM protein was identified in cattle [122,123], and after that, in rabbit and sheep [114]. Bovine and ovine TRIM5α have also demonstrated antiviral activity to different retroviruses. In contrast, the feline genome does not encode functional TRIM5α or TRIMCyp proteins [124].

The high genetic heterogeneity found within the capsid region is likely a consequence of a long-lasting direct contact with TRIM5α proteins. It is now clear that TRIM5 has been co-evolving with retroviruses for tens of millions of years. This long-term, “evolutionary arms race” has originated functional innovations in TRIM5α, including short insertions/deletions and tandem duplications, rapid sequence mutations and elevated levels of nonsynonymous substitution, balanced polymorphisms, and structural variation, including expansions of gene copy number and exon shuffling/trapping. The PRYSPRY domain of TRIM5α, the major determinant of viral specificity, shows extreme rates of positive selection within short stretches of this domain in primates [125], as well as in sheep [114]. The TRIM family is vigorously engaged in gene duplication [126], and in some mammalian genomes, expansions, inversions, and deletions of the *TRIM5* locus itself are evident [127]. Given the abundant evidence showing that retroviral pathogens have been ubiquitously present along primate evolution [128,129], and the variety of retroviruses that are known to colonize extant primates [130], it is reasonable to hypothesize that TRIM5α has long played, and continues to play, a role in governing patterns of susceptibility to cross-species transmission and spread (within species) of retroviral pathogens [131].

The evolutionary analysis of TRIM5 holds transposable lessons to the study of other intrinsic antiviral factors.

#### 6.1.1. *SRLV*

SRLV are restricted by sheep TRIM5α when overexpressed in cell culture, but the mechanism by which SRLV avoids this restriction *in vivo* is unknown [114]. Comparison of TRIM5α sequences revealed greater variation between caprine and ovine TRIM5α proteins than between ovine sequences, with the PRYSPRY being the most variable domain. Such variation was higher than expected, given that sheep and goats diverged 6 million years ago [132], whereas humans and chimpanzees, which encode more highly related TRIM5α sequences, diverged 7 million years ago [133]. The relative close-relatedness between sheep and goats is consistent with the ability of sheep (VMV) and goat (CAEV) lentiviruses to infect both ruminant species [134]. The high variability of both PRYSPRY [135] and CA of SRLV may account for the evolution of both virus and host, involving TRIM5α and CA interactions, as described for primate lentiviruses [136].

#### 6.1.2. *EIAV*

EIAV has provided a clear example of heterologous restriction in the case of TRIM5α. EIAV infection is restricted by rhesus TRIM5α, independent of either of the RING functions [113,137]. This is consistent with the retention of lentivirus restriction activity for TRIM5α variants completely lacking the RING domain. Perhaps, as discussed above, features of the lentivirus conical capsid diminish the requirement for RING domain function during the restriction process [113].

#### 6.1.3. *FIV*

Feline TRIM5α is incapable of restricting retroviruses, including *Murine leukemia* virus (MLV), HIV-1, or SIV of macaques (SIVmac) [138]. *Feliformia* express a truncated TRIM5 gene, a fact that explains why feline cells do not show a TRIM5-typical restriction to retroviruses (Figure 5). The feline mRNA of TRIM5 contains a premature stop codon expressing a RBCC protein without the B30.2/PRYSRPY domain. In cats, the missing PRYSPRY domain is not replaced by cyclophilin A (CypA) (TRIMCyp), as seen in some monkeys [138]. However, the truncated feline TRIM5 protein may be involved in LPS-mediated signaling, similarly to the human TRIM5α, potentially explaining why this gene is retained in Feliformia. Interestingly, a synthetic fusion of the feline TRIM5 to the feline CypA generated a potent inhibitor of FIV and HIV-1 [139], supporting that the RBCC domains of feline TRIM5 retain their intrinsic antiviral function [112].

#### 6.1.4. *BIV*

Bovine TRIM5α proteins inhibit the *in vitro* infection by several lentiviruses, such as FIV, SIV, and HIV; however, they do not inhibit the infection by BIV or have a minimal effect on the infection by EIAV. A similar situation applies to primate TRIM5α proteins, which, at best, partially restrict their related lentiviruses. As mentioned, retroviruses have evolved capsids that are only moderately restricted by the TRIM protein(s) expressed by the natural host [122].

### 6.2. APOBEC3

**Figure 6:**
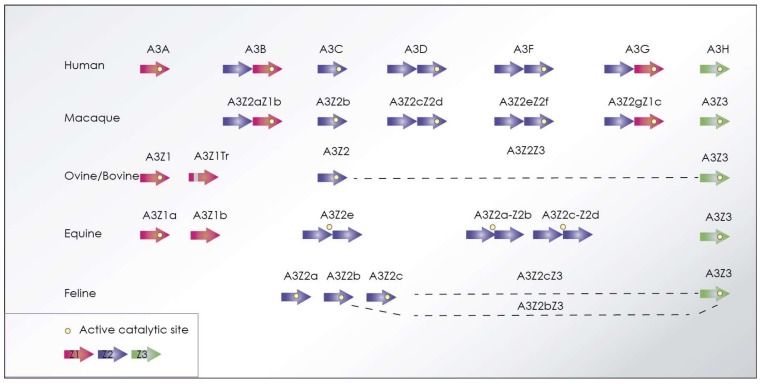
APOBEC3 proteins encoded by primates in comparison with ovine, bovine, equine, and felines highlighting Zn^2+^ domains and deaminase catalytic site. Ovine, bovine, and feline encode alternative splicing derived proteins (highlighted with a discontinuous line) and a truncated isoform in the case of small ruminants.

In recent years, much attention has been paid to the APOBEC3/Vif system, prompted by observations in the human immunodeficiency virus. The APOBEC3 proteins (also abbreviated as A3) belong to the *AID/APOBEC* gene family, only present in mammalian genomes. A3 proteins share a characteristic zinc (Z)-coordinating catalytic motif (Figure 6), and are able to mutate the viral genome resulting in disruption of the viral cycle.

The A3 proteins appear to exert their inhibitory activity mostly through a deaminase-dependent mechanism. A3 proteins produce deamination of C in reverse-transcribed first strand DNA, leading to G-to-A mutations in the positive strand of proviral DNA. A3 can be incorporated into progeny virions in producer cells (passenger A3) and inhibit lentiviral replication in the following replication cycle in target cells. A3 may restrict retroviruses in a deaminase-independent manner, such as *Friend murine Leukemia* virus [140], HIV [141], parvoviruses [142], parvo adeno-associated viruses [143], *Moloney murine leukemia* virus [144], SRLV [145], and hepatitis B virus [146,147,148]. Deaminase-independent mechanisms still remain elusive, and involve retrotranscription inhibition, impaired tRNA priming, and reduced RT processability [149].

In response to this, the lentiviral viral infectivity factor (Vif) is capable of restoring viral infectivity by triggering the degradation of most A3 proteins. The mechanism of A3 degradation is through ubiquitin- and proteasome-dependent pathways, by recruiting certain cellular proteins to construct a Vif-mediated E3 ubiquitin ligase complex. The cellular factors recruited include the scaffold protein Cullin, and the substrate adaptors Elongin B and Elongin C (ELOC). In addition, core-binding factor subunit beta (CBF-β) has been shown to be important for the primate lentiviral Vif function [150] and to form and stabilize the E3 ligase complex. Instead, non-primate lentiviruses employ CYPA as the co-factor to form the complex that will induce A3 polyubiquitination and degradation, thereby suppressing A3-mediated antiviral activity.

Recent investigations have suggested that lentiviral *vif* genes evolved to combat mammalian APOBEC3 proteins, and have further proposed that the Vif–A3 interaction may help to determine the co-evolutionary history of cross-species lentiviral transmission in mammals [151]. In most cases, the interaction of Vif with A3 proteins is species-specific [152], but some lentiviral Vif proteins are capable of triggering the degradation of A3 proteins of several other mammals in addition to their natural host species [153]. 

#### 6.2.1. *SRLV*

VMV Vif is essential for the infection of primary macrophages and for *in vivo* infections, highlighting the importance of APOBEC3 restriction in natural infection. Sheep (*Ovis aries*) encode three different A3 genes: *APOBEC3Z1* (A3Z1), *APOBEC3Z2* (A3Z2), and *APOBEC3Z3* (A3Z3), that result in the synthesis of a fourth protein (A3Z2Z3) due to alternative splicing. Ovine A3Z2-Z3, containing two Zn-domains, is able to restrict SRLV infection, as well as HIV-1 infectivity [153]. The cellular proteins recruited by SRLV Vif in sheep include Cullin5 (CUL5), and the substrate adaptors Elongin B/C25 and cyclophilin A (CYPA, also known as peptidylprolyl isomerase A), as cofactors for degrading sheep APOBEC3.

Unlike primate lentiviruses, SRLV Vif does not interact with CBF-β [150]. The mechanism involving CYPA implies that VMV Vif hijacks this molecule as a cofactor to reconstitute the E3 ligase [154]. Despite the ability of SRLV Vif to degrade A3Z2Z3, circumventing this defense mechanism, infection can be controlled under specific *in vivo* and *in vitro* conditions. A3Z1 is resistant to Vif degradation, and is highly expressed in resistant cells, such as monocytes and M1-macrophages [155]. Furthermore, A3Z1 can be incorporated into virions, even in the presence of viral Vif. *In vitro* expression of A3Z1 reduced the production of SRLV and the infectivity of HIV-1, most likely through deaminase mechanisms.

#### 6.2.2. *EIAV*

EIAV is genetically the simplest virus within the genus, and contains only three accessory genes, namely *tat*, *rev*, and *S2*, lacking *vif*, a unique feature of EIAV. This is of particular interest concerning the restriction exerted by APOBEC3 proteins. The ability of human A3G to inhibit EIAV replication is a clear example of restriction of heterologous viruses [156]. Also, human A3F, murine A3Z2Z3, and porcine A3Z2Z3 exert antiviral activity against EIAV [157].

Horses express six different A3 genes with single or double cytidine deaminase domains [158] (Figure 6). This is close to the quantity present in the human genome, and greater than that reported in any other non-primate species. While being potent inhibitors of heterologous retroviruses through deamination, equine A3s exert weak restriction against EIAV, despite efficient incorporation into virions. Only A3Z3 demonstrated effective restriction of EIAV replication in CrFK cells [157]. This suggests that EIAV has evolved to develop a novel mechanism that acts after virion incorporation to escape antiviral activities of APOBEC3 proteins in the absence of Vif. Whether EIAV persists without any countermeasure against the activity of the equine A3 proteins remains unknown. Zielonka et al. [157] propose that by tolerating the incorporation of inactive equine A3 proteins (A3Z1b, A3Z2a-Z2b, and A3Z2e), EIAV avoids the encapsidation of active antiviral A3Z3 by adapting its cellular tropism to macrophages, in which A3Z3 expression is very low. However, as raised in human and ovine cases, restriction exerted by the different A3 proteins may be highly influenced by the cellular type in which restriction is evaluated, A3Z1 being inactive in dividing cells, such as 293-T or TIGEF, but exhibiting antiviral activity in myeloid cells [144,155].

In addition to cytidine deaminases, adenosine deaminases that act on RNA also have a role in restricting or promoting viral replication, being highly dependent on the virus. Equine ADAR1 is a positive regulator of EIAV replication by promoting LTR and Rev Responsive Element (RRE) activities [159], similar to human ADAR1 [160].

#### 6.2.3. *FIV*

As in other species, feline T-cells and macrophages express APOBEC3. Feline *A3* gene encoding a Z1 domain protein does not exist but, as in the human case, three similar copies of *Z2* genes (*A3Z2a*, *A3Z2b*, and *A3Z2c*), a single copy of *Z3* (*A3Z3*), and also an A3Z2-Z3 by read-through transcription and alternative splicing, are found (Figure 6).

FIV Vif protein induces the degradation of feline A3 proteins, by interacting with CUL5, ELOB, and ELOC [152,161], but not with CBF-β [150]. FIVΔvif is moderately inhibited by feline A3Z3 and is strongly suppressed by feline A3Z2-Z3. This antiviral activity against FIVΔvif is correlated with the detection of cytidine deamination. It is unknown why the feline A3Z2 proteins do not inhibit FIVΔvif or FIV despite efficient deaminase activity [112]. The interaction between Vif and A3 proteins is Zn-independent [152].

The Vif protein from domestic cat FIV can efficiently inhibit A3 of other Felidae (puma, lion, lynx, bobcat, or tiger) [162]. These results indicate that A3 proteins from big cats are not major determinants that prevent cross-species transmission of FIV from the domestic cat to these closely related animal species. A characterization of the molecular interaction of domestic cat FIV Vif, with A3s of different felids, may reveal the interacting domains of Vif and A3, besides explaining the broad activity of domestic cat FIV Vif. However, differences are seen, as *puma lentiviruses group A* (PLV-A) counteract the antiviral action of A3Z3 of both puma and bobcat, whereas PLV-B Vif counteracts only puma A3Z3. The amino acid at position 178 in the puma and bobcat A3Z3 is exposed on the protein surface, and determines the sensitivity to PLV-B Vif-mediated degradation [151].

#### 6.2.4. *BIV*

As with sheep, cattle (*Bos taurus*, Bt) encode three *A3* genes: *btA3Z1*, *btA3Z2*, and *btA3Z3*, and another splicing variant, *btA3Z2Z3* [163]. Only A3Z3 and A3Z2Z3 exhibit a strong anti-lentiviral ability in *in vitro* cell culture systems [146] that is counteracted by the BIV and JDV Vif proteins which degrade cattle A3 proteins [44].

Unlike other lentiviruses, BIV Vif interacts with Cullin 2 (CUL2), ELOB/C, RBX1, but not with CBF-β, CypA, or CUL5, to form a Cullin–RING ubiquitin ligase (CRL) complex, and degrade bovine A3 proteins (A3Z2Z3 and A3Z3) [154,164]. In addition, primate lentiviral Vif binds CUL5-RBX2, whereas BIV Vif interacts with CUL2-RBX1. BIV Vif and JDV Vif are the only known retroviral proteins that can interact with CUL2 [164]. Similar to BIV, JDV Vif hijacks CUL2, ELOB/C, and RBX1, also without the need for CBF-β, to form E3 ubiquitin ligase and induce the degradation of the btA3 proteins [163].

As neither SRLV, FIV, BIV, nor JDV Vif require CBF-β for degrading APOBEC3 proteins, it has been suggested that non-primate lentiviral Vif induces APOBEC3 degradation through a different mechanism [150].

Bovine A3Z1, as well as human and ovine counterparts, is resistant to BIV and JDV Vif-mediated degradation [163,164] and display no restriction on viral replication in non-myeloid cells. This result suggests that A3Z1 evolved to escape from Vif-mediated degradation, which might allow it to perform other important biological functions. A3Z3 of gaurs (*Bos gaurus*), another bovid species living in Asia, has been shown to be resistant to JDV Vif-mediated degradation [44]. Both resistances support the coevolution of bovine and their lentiviruses [163].

### 6.3. Tetherin

Tetherin or BST2 (bone marrow stromal cell antigen 2, also called CD317, HM1.24) is a type I and II interferon-induced transmembrane protein, first discovered as a cellular restriction factor against HIV-1 infection [165]. Later, tetherin has been found to inhibit the replication of several enveloped viruses by blocking viral release, showing very broad antiviral activities against retroviruses, filoviruses, arenaviruses, paramyxoviruses, herpesviruses, and rhabdoviruses [166]. During the late phase of viral replication, BST2 causes nascent viruses to remain trapped at the surface of the infected cell, and to accumulate, thereafter, in endosomes following internalization [167].

Tetherin/BST2 is an unusual type II integral membrane protein, with four structural domains: an N-terminal cytoplasmic tail, a single transmembrane domain, an extracellular CC domain, and a C-terminal glycosylphosphatidylinositol (GPI) lipid anchor [112,168]. The antiviral activity of tetherin seems to be related to this modular structure [169]. It can anchor into viral membranes, trapping enveloped viral particles on the surface of infected cells, to be internalized and degraded [170]. This is done either by direct crosslinking or by the formation of dimers between adjacent coiled-coil domains [167].

Tetherin is not well conserved across species, so it inhibits viral replication in a species-specific manner [166]. In humans, tetherin is expressed in several specialized cell types, such as hepatocytes, monocytes, epithelial cells, terminally differentiated B-cells, and bone marrow stromal cells. It is upregulated in many cell types upon treatment with interferon [112].

**Figure 7:**
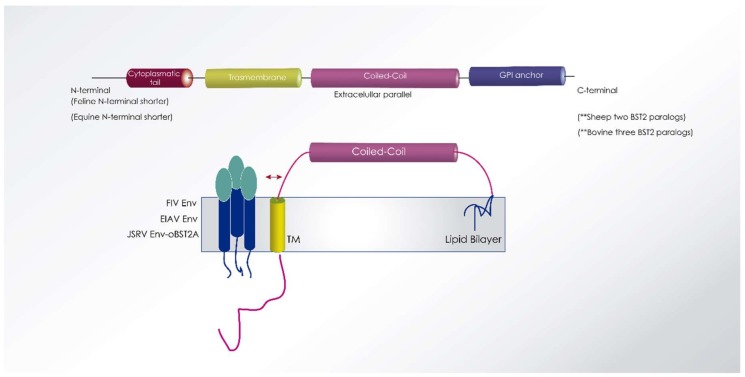
BST2/tetherin protein structure representation including animal lentiviruses targeted species. Cytoplasmatic tail, transmembrane, coiled-coil, and GPI anchor domains are represented. The N-terminal cytoplasmatic tail of feline and equine tetherin is characterized by a shorter region compared to other species. Sheep and bovine tetherin have two and three paralogs, respectively, which inhibit virus infectivity by different mechanisms [171].

Lentiviruses have developed methods to antagonize the effect of tetherin. While primate lentiviruses encode particular accessory proteins (Vpu, Vpx), other non-primate lentiviruses countermeasures rely mostly on the envelope glycoproteins [11] (Figure 7).

#### 6.3.1. *SRLV*

Ovine tetherin/BST2A impedes viral exit of *Jaagsiekte sheep* retroviruses (JSRV) while ovine BST2B isoform displays a novel antiviral activity impairing the normal cellular trafficking of JSRV envelope glycoproteins [172] (Figure 7). Both ovine tetherins show low identity with human (~35%), bovine (~60%) or equine (~30%) tetherins, while BST2A isoform was similar to deer (76%) and fallow deer (72%) tetherins [173].

Studies describing ovine BST2 antiviral mechanisms against SRLV are not available so far. However, activity has been suggested to play a role in natural restriction *in vivo* [174] as well as in preserving species barrier in deer and fallow deer [173].

#### 6.3.2. *EIAV*

Equine tetherin shares 53%, 40%, 36%, and 34% amino acid sequence identity with feline, human, simian, and murine tetherins, respectively. Like feline tetherin, equine tetherin has a shorter N-terminal domain than human tetherin (Figure 7). Equine tetherin is expressed in macrophages, fibroblasts, and other equine cell types, localized on the cell surface, and strongly blocks HIV-1, SIV and EIAV release from virus-producing cells. The antiviral activity of equine tetherin is neutralized by EIAV envelope protein, but not by the HIV-1 accessory protein Vpu, which is a human tetherin antagonist. Similarly, EIAV envelope protein does not counteract human tetherin. Unlike Vpu, EIAV Env protein does not degrade equine tetherin despite efficient binding by an as-yet undescribed mechanism [166].

Attenuated EIAV strains for vaccination or for immunological studies are classically obtained through different passage attenuation systems, generally in cells from donkey [175]. This, together with the lower viral loads and milder disease observed in donkeys, prompted studies on tetherin from horses and donkeys. Donkey tetherin is shorter, and differs from that of horse in the transmembrane and ectodomains, but displayed similar antiviral activity against EIAV and HIV-1, and equally activated NF-κB signaling. This suggests that both equine tetherins orthologues have evolved independently after speciation [166]. Donkey tetherin has different antiviral effects depending on the virulence of the challenging strains [175]. A shorter isoform of ovine tetherin also displays stronger antiviral activity than that of full-length [171].

#### 6.3.3. *FIV*

Tetherin is expressed in many feline cell lines, and expression is induced by interferons, including IFN-α, IFN-ω, and IFN-γ [176] and displays potent inhibition of FIV and HIV-1 particle release. The N-terminal cytoplasmic tail of feline BST2 (fBST2) is characterized by a shorter N-terminal region compared to those of other known homologs, with an amino acid sequence identity of 44.4% compared to human tetherin [167,177]. The addition of a peptide in the cytoplasmic tail region of fBST2 may influence its antiviral activity. The extracellular domain of feline tetherin/BST-2 has two putative *N*-linked glycosylation sites: N79 and N119. Complete loss of *N*-linked glycosylation by introduction of mutations into both sites resulted in almost complete abolition of its antiviral activity [178]. In contrast to human BST2, the wild-type fBST2 did not show the ability to activate NF-κB [167].

Tetherin is antagonized by the FIV Env protein, but unlike other tetherin antagonists, FIV Env cannot act in *trans* to rescue Vpu-deficient HIV-1 [178,179]. It must be incorporated specifically into FIV virions to be active [180]. Also, unlike other retroviral antagonists, but similar to *Ebola* virus Env, it does not act by reducing intracellular or cell surface tetherin levels. FIV Env might exclude tetherin locally or by direct assembly to tetherin-negative membrane domains. Other distinctive features are apparent, including evidence that FIV evolved an equilibrium in which tetherin is both a restriction factor and a cofactor, since tetherin is required for optimal particle release from cells [180]. Lastly, while tetherin may prevent the release of nascent viral particles, cell-to-cell spread remains efficient, and tetherin upregulation may enhance syncytium formation in the presence of abundant viral receptors. Accordingly, tetherin expression *in vivo* may promote the selective expansion of viral variants capable of more efficient cell-to-cell spread [139].

#### 6.3.4. *BIV*

Three isoforms of bovine BST2 have been described: bBTS2A1, bBTS2A2, and bBTS2B [181,182]. Like human BST2, bBST2A1 suppresses bovine retrovirus release (*bovine leucosis* and *bovine foamy* viruses, BLV and BFV) but it is inactive against cell-to-cell infection of BIV and BFV, similar to what has been reported for feline tetherin and FIV [179]. However, human BTS2 inhibits the cell-to-cell infection of these viruses [182].

All these data suggest that BST2 from different species may possess similar activity on virus release, but differ in their effect on virus cell-to-cell transmission.

## 7. Other Host Protein Factors

### 7.1. SAMHD1

Recently, the human protein SAMHD1 has been identified to be causative for the post-entry restriction of HIV-1 in myeloid cells [183,184]. Viral particle-associated Vpx protein induces a proteasome-dependent degradation of SAMHD1 in the target cell very early after entry. The SAMHD1 protein has two domains that are widely found in all genomes: an N-terminal SAM domain followed by an HD domain. SAM (sterile α motif) domains have diverse functions, such as binding to kinases, other proteins, or RNA [185]. The HD domain with histidine and aspartic acid residues for metal coordination defines a superfamily of metal-dependent phosphohydrolases, which includes many proteins that are involved in nucleic acid metabolism, such as dGTPases, nucleotidyltransferases, and helicases.

The antiviral mechanism of SAMHD1 has not yet been identified, but SAMHD1 may exert antiviral activity through degradation of viral RNA, rather than through its dNTPase activity [186,187]. However, controversy has been raised related to these findings [188,189]. Recent findings suggest that innate immune responses are downregulated by SAMHD1 due to NF-κB, and type-I IFN inhibition [190] and interference with HIV replication cycle may also involve LTR promoter activity [191].

Studies based on SAMHD1 antiviral activity regarding non-primate lentiviruses are currently lacking. It would be interesting to determine whether the feline gene for SAMHD1 (found on cat chromosome A3) encodes an antiviral protein, and whether FIV expresses a viral counteracting measure against feline SAMHD1 that functions similarly to Vpx. FIV possesses an additional small open reading frame, termed *orfA*, which encodes a factor that facilitates the transactivation of transcription during viral replication [192].

### 7.2. HEXIM1

Tat proteins from BIV, JDV, HIV-1, HIV-2, SIV, and EIAV bind to the *TAR* (transactivation region) present at the 5′ end of viral RNA transcripts and recruit the cellular positive transcription elongation factor b (P-TEFb), which consists of the cyclin-dependent kinase 9 (CDK9) and cyclin T1, to the viral promoter. The hexamethylene bisacetamide (HMBA)-inducible protein 1 (HEXIM1) is a component of the inactive positive transcription elongation factor (P-TEFb) [193]. Upon binding to the 7SK small nuclear RNA, HEXIM1 oligomers undergo a conformational change and then bind to cyclin T1. Following association with the P-TEFb complex, HEXIM1 inhibits the activity of CDK9 [194]. Through this mechanism, human HEXIM1 suppresses HIV-1 Tat transactivation [195]. Similarly, the bovine HEXIM1 (BHEXIM1) inhibits Tat-mediated BIV LTR transcription by competing with Tat for binding to B-cyclin T1 [196]. Based on these results, these authors proposed that BHEXIM1 would have a potential role on the BIV latent lifecycle, and the absent clinical signs of infected livestock.

### 7.3. Schlafen 11

SLFN11 is a novel restriction factor also induced by type-I interferon, based on bias regarding relative synonymous codon usage by binding to tRNA.

Among non-primate lentiviruses, Schlafen 11 has been studied in the EIAV field so far. Overexpression of equine SLFN11 inhibited EIAV replication, and silencing increased viral production by a mechanism involving impairment of viral mRNA translation [197].

## 8. Concluding Remarks

Recent research on restriction factors involved in lentivirus biology is offering promising results in terms of antiviral activity. By overexpressing them exogenously as a potential treatment or rather by selecting highly responder individuals or those harboring advantageous polymorphisms, new strategies in the control of non-primate lentiviruses may be envisioned.

However, care should be taken in these new designs, since dysregulation of the pathways involved by exogenous administration may lead to IFN-α overproduction and disease development due to onset of autoimmune processes.

New restriction factors are being discovered, and further studies are needed to investigate, for example, the role, if any, of HEXIM1 in lentiviruses lacking Tat protein, or the importance of specific polymorphisms in *TLR* genes associated with lentivirus resistance.

In an attempt to relieve adverse effects related to interferon-based therapies, *in vivo* testing of restriction factors as therapeutic agents is being conducted in the human field [198] and its application to the veterinary field may contribute to both animal and human health.
